# The *Escherichia coli* Phospholipase PldA Regulates Outer Membrane Homeostasis via Lipid Signaling

**DOI:** 10.1128/mBio.00379-18

**Published:** 2018-03-20

**Authors:** Kerrie L. May, Thomas J. Silhavy

**Affiliations:** a Department of Molecular Biology, Princeton University, Lewis Thomas Laboratory, Princeton, New Jersey, USA; National Cancer Institute

**Keywords:** cell signaling, Gram-negative bacteria, lipopolysaccharide, outer membrane, phospholipase

## Abstract

The outer membrane (OM) bilayer of Gram-negative bacteria is biologically unique in its asymmetrical organization of lipids, with an inner leaflet composed of glycerophospholipids (PLs) and a surface-exposed outer leaflet composed of lipopolysaccharide (LPS). This lipid organization is integral to the OM’s barrier properties. Perturbations of the outer leaflet by antimicrobial peptides or defects in LPS biosynthesis or transport to the OM cause a compensatory flipping of PLs to the outer leaflet. As a result, lipid asymmetry is disrupted and OM integrity is compromised. Recently, we identified an *Escherichia coli* mutant that exhibits aberrant accumulation of surface PLs accompanied by a cellular increase in LPS production. Remarkably, the observed hyperproduction of LPS is PldA dependent. Here we provide evidence that the fatty acids generated by PldA at the OM are transported into the cytoplasm and simultaneously activated by thioesterification to coenzyme A (CoA) by FadD. The acyl-CoAs produced ultimately inhibit LpxC degradation by FtsH. The increased levels of LpxC, the enzyme that catalyzes the first committed step in LPS biosynthesis, increases the amount of LPS produced. Our data suggest that PldA acts as a sensor for lipid asymmetry in the OM. PldA protects the OM barrier by both degrading mislocalized PLs and generating lipid second messengers that enable long-distance signaling that prompts the cell to restore homeostasis at a distant organelle.

## IMPORTANCE

The outer membrane of Gram-negative bacteria is an effective permeability barrier that protects the cell from toxic agents, including antibiotics. Barrier defects are often manifested by phospholipids present in the outer leaflet of this membrane that take up space normally occupied by lipopolysaccharide. We have discovered a signaling mechanism that operates across the entire cell envelope used by the cell to detect these outer membrane defects. A phospholipase, PldA, that functions to degrade these mislocalized phospholipids has a second, equally important function as a sensor. The fatty acids produced by hydrolysis of the phospholipids act as second messengers to signal the cell that more lipopolysaccharide is needed. These fatty acids diffuse across the periplasm and are transported into the cytoplasm by a process that attaches coenzyme A. The acyl-CoA molecule produces signals to inhibit the degradation of the critical enzyme LpxC by the ATP-dependent protease FtsH, increasing lipopolysaccharide production.

## INTRODUCTION

The outer membrane (OM) of Gram-negative bacteria is a formidable permeability barrier that enables the uptake of essential nutrients and protects the cell against host and environmental assaults. An important feature of this protective shell is the unique asymmetrical organization of lipids. While the inner leaflet is composed of glycerophospholipids (PLs), the surface-exposed outer leaflet consists almost exclusively of lipopolysaccharide (LPS) (1, 2). The tripartite structure of LPS consists of lipid A, a core oligosaccharide, and an extracellular O-antigen polysaccharide chain, although the O-antigen component is not synthesized in *Escherichia coli* K-12 (3, 4). Divalent cations also contribute to OM integrity by facilitating strong lateral electrostatic interactions between negatively charged phosphates in adjacent LPS molecules (5–8). Maintenance of the integrity of this outer leaflet is critical to protect the cell against bile salts, detergents, antibiotics, and antimicrobial peptides (5, 9). Perturbation of the outer leaflet of the OM due to defective LPS biosynthesis or transport or exposure to antimicrobial peptides or chelators leads to a compensatory accumulation of PLs in the outer leaflet (5, 10, 11). As a result, lipid asymmetry is disrupted and OM integrity is compromised. In *E. coli*, several systems respond to lipid asymmetry; the Mla system facilitates retrograde PL trafficking (12–14), the OM phospholipase PldA processively degrades surface PLs (9, 15, 16), and the palmitoyltransferase enzyme PagP transfers a palmitate chain from mislocalized phospholipids to lipid A in the OM (5, 10, 11).

The Mla (maintenance of lipid asymmetry) system is composed of components in each compartment of the cell envelope, such that it can facilitate retrograde transport of mislocalized phospholipids from the outer leaflet of the OM to the inner membrane (IM) (12–14). If any of the *mla* genes (*mlaA*, -*B*, -*C*, -*D*, -*E*, and -*F*) are disrupted, OM permeability defects, including increased sensitivity to bile salts and detergents, can be observed, even under normal laboratory growth conditions (12).

OM phospholipase A (PldA) is an integral OM protein. The active form of this enzyme is a homodimer of β-barrel subunits (16–18) that uses calcium as a cofactor (19, 20) to form a substrate-binding pocket positioned at the cell surface, which enables this enzyme to catalyze the hydrolysis of acyl ester bonds in phospholipids and lysophospholipids at the outer leaflet of the OM (15, 16, 19).

The ability of *pldA* overexpression to functionally complement the inactivation of any *mla* gene and the synergistic defects that occur upon the loss of both systems clearly demonstrate the functional overlap between the PldA phospholipase and the Mla pathway in the removal of mislocalized phospholipids (12). It appears that PldA and Mla act at the OM as quality control systems for lipid asymmetry; however, whether the cell can sense these lipid perturbations and respond further to restore homeostasis is unclear.

Recently, we identified a dominant mutation, mlaA* (*mlaA*_ΔN41-F42_), in the gene that encodes the OM lipoprotein component of the Mla system (12, 13, 21). This mutation not only inactivates the Mla system but actively disrupts lipid asymmetry, triggering aberrant accumulation of PLs on the surface of the OM (21). Recent structure determination shows that MlaA is a donut-shaped molecule that is embedded in the inner leaflet of the OM (13). This structure allows PLs located in the outer leaflet, but not PLs located in the inner leaflet, to enter a central amphipathic pore for delivery to the periplasmic component MlaC. The mlaA* mutation likely disrupts an α-helix that runs parallel to the membrane, disrupting the donut shape and allowing PLs from the inner leaflet to enter the pore and flow into the outer leaflet, a process driven by mass action (13).

Intriguingly, this increase in PLs in the outer leaflet causes a corresponding and detrimental increase in LPS production (21). The resultant membrane destabilization and massive loss of OM lipids via vesiculation produce a characteristic cell death phenotype in stationary phase when divalent cations and energy are limited. Cytoplasmic contraction due to the net flow of lipids from the IM to the OM occurs in an attempt to compensate for this catastrophic lipid loss. IM rupture and cell death can be suppressed by providing an energy source (e.g., glucose) to allow the synthesis of more lipid or by stabilizing the OM to prevent lipid loss, either by supplementation with divalent cations or by preventing LPS hyperproduction. Surprisingly, this mlaA*-dependent cell death is also suppressed by loss of the OM phospholipase PldA. PldA activation by mlaA* is not surprising since this mutation facilitates aberrant accumulation of surface PLs known to trigger PldA activation. However, it was unexpected that the elevated LPS production requires PldA activity. Moreover, in the absence of PldA activity, LPS is restored to the wild-type (WT) level despite the fact that OM asymmetry is exacerbated. This effect is specific to PldA activity, as loss of PagP has no effect on cell death (21).

Here we show that the OM phospholipase PldA constitutes the sensor of a novel signaling pathway that detects PL accumulation in the outer leaflet of the OM and signals the cytoplasm to increase LPS production as part of the cellular response to disruption of asymmetry.

## RESULTS

### mlaA* triggers a hyperproduction of LPS that is dependent on the OM phospholipase PldA.

**FIG 1:**
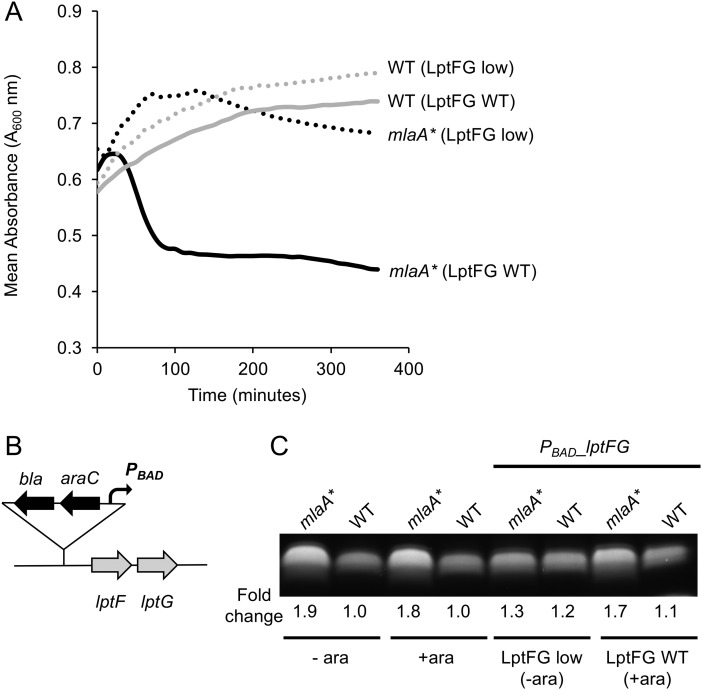
mlaA* mutants lyse because of hyperproduction of LPS. (A) Cells were cultured with or without arabinose (LptFG WT and LptFG low, respectively) to modulate *lptFG* expression and thus LPS transport. Cells were then transitioned to spent medium, and cell density was monitored (*A*_600_). The data show that limitation of *lptFG* expression suppressed cell death caused by the mlaA* mutation. (B) Schematic showing the *bla*-*araC*-*P*_BAD_ cassette that was introduced into the strains so that *lptFG* expression could be regulated by an arabinose-inducible promoter (22). (C) Analysis of whole-cell LPS levels indicated that when expression of *lptFG* in mlaA* mutant cells was limited (LptFG low), LPS levels were lower than those in mlaA* mutant strains with efficient *lptFG* expression (LptFG WT).

**FIG 2:**
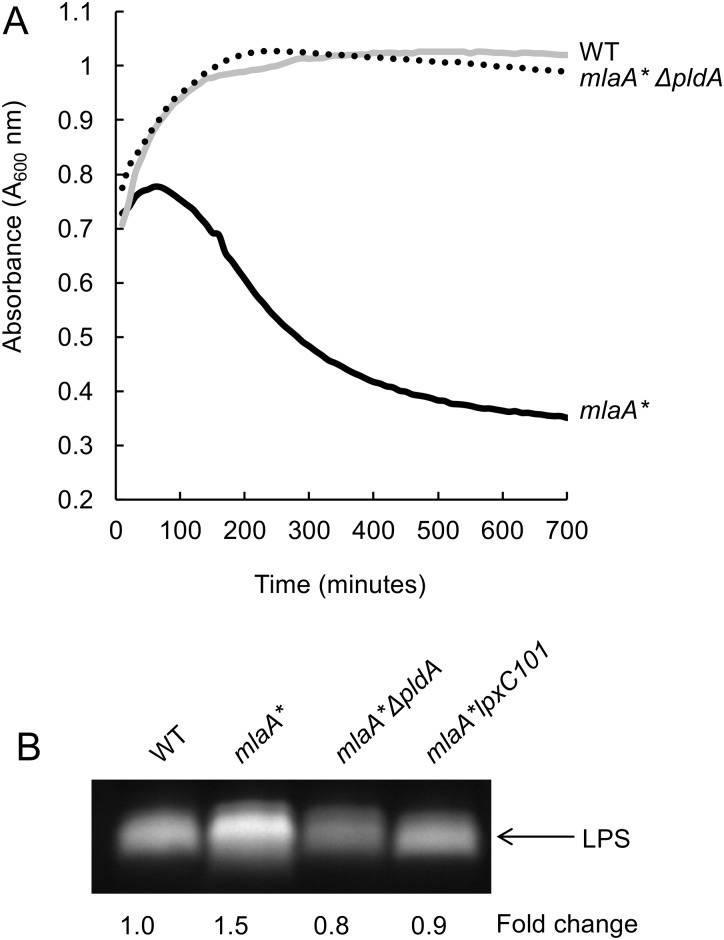
mlaA*-induced cell death and hyperproduction of LPS are dependent on the OM phospholipase PldA. (A) Loss of PldA suppressed cell death in the spent-medium transition assay (as monitored by changes in cell density [*A*_600_]). (B) Loss of PldA also suppressed mlaA*-dependent LPS hyperproduction.

We previously showed that the mlaA* mutation triggers aberrant accumulation of PLs in the outer leaflet, leading to hyperproduction of LPS and cell death in spent medium (21). To examine if simply limiting LPS transport to the OM can suppress cell death caused by mlaA*, we engineered *E. coli* strains that enable tunable expression of components of the LPS transport pathway. We used generalized P1 transduction to introduce DNA containing *lptFG* under the control of an arabinose-inducible promoter (22) (Fig. 1B). In the presence of arabinose, these strains produce LptF and LptG in sufficient amounts (comparable to WT amounts) to promote efficient LPS transport. However, in the absence of arabinose, LptF and LptG become limiting and LPS transport is decreased. We cultured our engineered strains to the mid-exponential growth phase with or without arabinose and then transitioned them to spent medium and monitored changes in cell density. Following the induction of *lptFG* with arabinose, mlaA* cells lysed upon a transition to spent medium, whereas limiting *lptFG* expression completely suppressed cell death (Fig. 1A). Interestingly, although the aim of this experiment was to limit LPS transport in the mlaA* mutant strain, it was evident that limiting expression of *lptFG* led to a corresponding decrease in LPS levels (Fig. 1C), suggesting that a mechanism may exist for the cell to reduce LPS production when LPS transport is inhibited. Nonetheless, these data clearly show that the mlaA* mutation causes a detrimental increase in LPS levels and that simply limiting LPS transport and/or synthesis is sufficient to prevent cell death in stationary phase and spent medium. Remarkably, this LPS hyperproduction and cell death were completely suppressed in the absence of the OM phospholipase PldA (21) (Fig. 2). Loss of PldA completely suppressed cell death in spent medium (Fig. 2A) and reduced LPS to WT levels (Fig. 2B). Collectively, these data suggest that in response to a disruption of OM lipid homeostasis, specifically, disruption of the integrity of the LPS outer leaflet due to aberrant accumulation of surface-exposed phospholipids, the cell responds by increasing LPS production in a PldA-dependent manner.

### Fatty acid uptake via FadD is required for a mlaA*-dependent increase in LPS production.

It was unclear how PldA activity at the OM might influence LPS production in the cytoplasm. There is increasing evidence that the biosynthetic pathways for LPS and phospholipids are regulated by levels of their fatty acid constituents (23). We reasoned that the by-products of PldA PL degradation might be transported to the cytoplasm and signal the cell to increase LPS production. PldA processively degrades surface-exposed phospholipids to produce lyso-PLs, fatty acid, and glycerophosphodiester by-products (15, 24). Cellular pathways exist to metabolize and transport each of these by-products into the cytoplasm. Therefore, each of these factors has the potential to signal in the cytoplasm to influence LPS biosynthesis. Lysophospholipids can be transported across the IM via the phospholipid flippase LplT (25, 26). Glycerophosphodiesters can be converted to G3P by the periplasmic glycerophosphodiester phosphodiesterase GlpQ (27) and then transported from the periplasm into the cytosol via the GlpT ABC transporter (28). Finally, exogenous or membrane-derived fatty acids can be transported to the cytoplasm and activated by the acyl coenzyme A (acyl-CoA) synthetase FadD (29–31).

**FIG 3:**
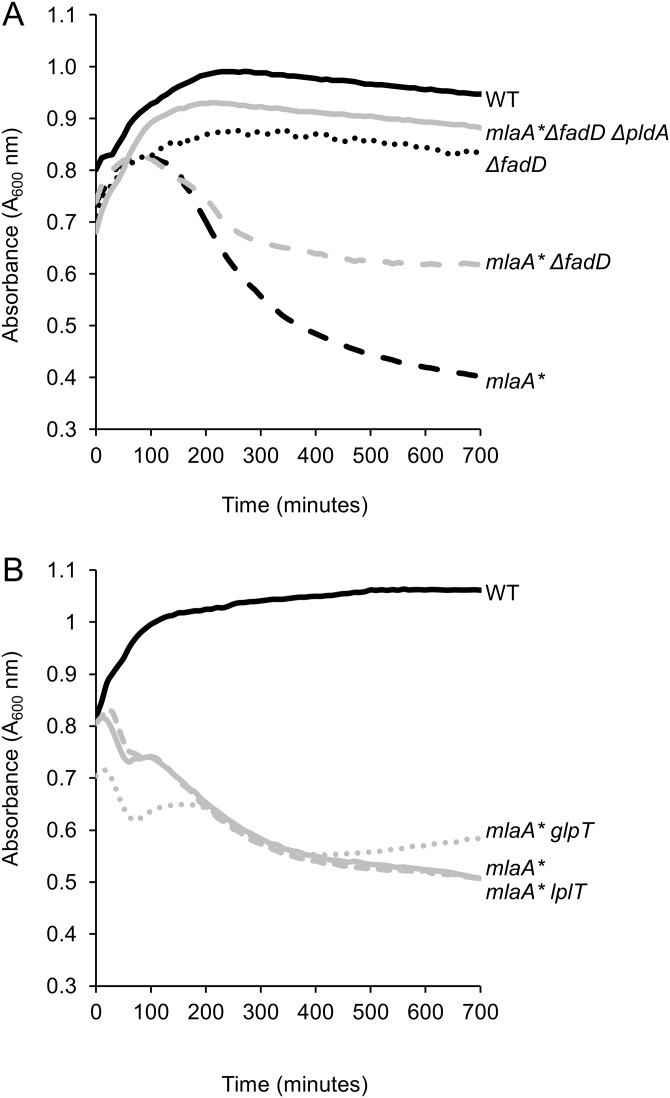
Blocking of FadD-dependent fatty acid transport mitigates mlaA* cell death. Cells were grown to mid-exponential phase and transitioned to spent medium, and changes in cell density (*A*_600_) were monitored over time. Loss of FadD suppressed cell death in the spent-medium transition assay (A), while loss of LplT or GlpT did not (B).

**FIG 4:**
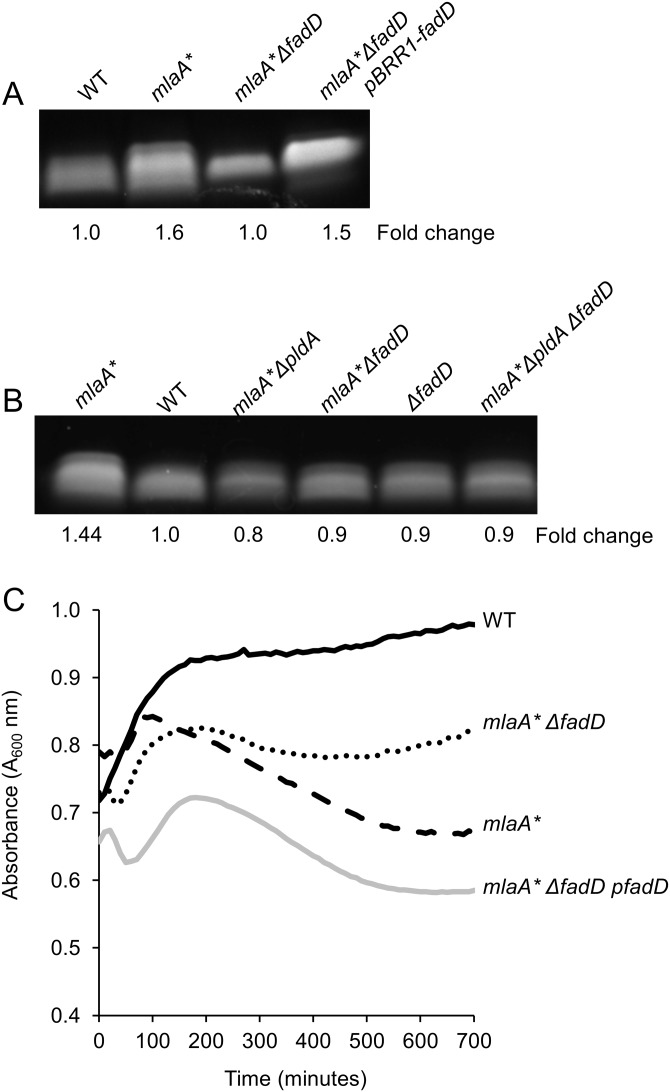
Blocking of FadD-dependent uptake of fatty acids prevents increased LPS production. Analysis of whole-cell LPS levels demonstrated that loss of *fadD* suppressed mlaA*-dependent LPS hyperproduction (A). Loss of *fadD* was equivalent to loss of *pldA* in preventing mlaA*-dependent LPS hyperproduction (B). Expression of the *fadD* open reading frame in *trans* demonstrated that suppression of LPS hyperproduction (A) and cell death in a spent-medium transition assay (C) were dependent on FadD, and not *sroD*, in a *fadD*-null strain.

We introduced null mutations to block the transport and/or recycling of the various PldA by-products and assessed mlaA*-dependent cell death in an overnight culture (see Table S2 in the supplemental material) and spent medium (Fig. 3). Most of the null mutations we examined had no effect on stationary-phase cell death. In particular, disruption of either G3P (*glpT*) or lysophospholipid (*lplT*) transport had no effect on cell death in spent medium (Fig. 3B). However, blocking FadD-dependent fatty acid transport and activation prevented the PldA-dependent LPS increase in mlaA* mutant cells and partially suppressed cell death (Fig. 3A and 4A). FadD typically acts downstream of the OM protein FadL (32, 33), which transports exogenous long-chain fatty acids across the OM; however, loss of *fadL* had no effect on mlaA*-dependent cell death (Table S2). The 3′ end of *fadD* encodes a small RNA, *sroD*, that has been shown to be expressed for a brief period at the onset of stationary phase (34). Since the null mutation of *fadD* also disrupted the region containing *sroD*, we expressed the open reading frame of *fadD* alone in *trans* to ensure that suppression of mlaA* phenotypes was entirely due to loss of FadD and not *sroD*. Indeed, expression of *fadD* alone was sufficient to restore LPS hyperproduction and cell death (Fig. 4A and C), confirming that *sroD* was not involved in PldA signaling.

### The mlaA*-dependent LPS increase and cell death are caused by PldA-dependent LpxC stabilization.

**FIG 5:**
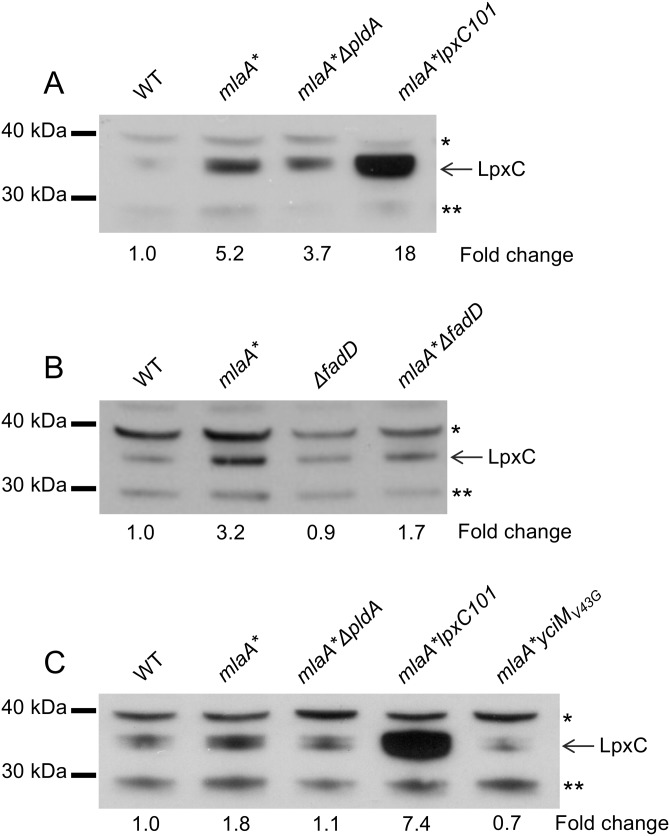
FadD-dependent uptake of PldA fatty acid by-products leads to increased LpxC levels. Anti-LpxC immunoblot assays of whole-cell LpxC levels demonstrated that the mlaA* mutation increased LpxC levels (A to C). Loss of *pldA* (A and C), loss *fadD* (B), and the *yciM*_V43G_ mutation (C) prevented mlaA*-dependent LPS hyperproduction. A strain harboring the *lpxC101* (*envA1*) (54) mutation, where LpxC levels are significantly elevated, was used as a positive control for LpxC migration (C). Samples equivalent to 5 × 10^7^ cells were loaded into each lane. Protein bands indicated by asterisks (*, **) represent cross-reactive species recognized by polyclonal anti-LpxC antiserum.

The lipid A and core components of LPS are synthesized at the interface between the IM and the cytoplasm (3). The equilibrium constant of the enzyme that catalyzes the first step in the biosynthetic pathway (performed by LpxA) is unfavorable (35), and it is the second reaction in the pathway, performed by the UDP-3-*O*-acyl-*N*-acetylglucosamine deacetylase LpxC, that is the first committed step in lipid A biosynthesis (36). We had previously shown that concurrent with the increased LPS production, increased cellular levels of LpxC could be observed (21). The increased LpxC production was due not to transcriptional upregulation (21) but likely to stabilization of the protein against proteolysis by the FtsH protease (37–39). We wanted to test whether the observed increases in LpxC were dependent on PldA and FadD, so we examined LpxC levels by Western blotting of whole-cell lysates. We found that loss of either PldA activity or FadD decreased mlaA*-dependent LpxC levels, thereby preventing increased LPS production (Fig. 5).

An integral IM adapter protein, YciM, has been shown to facilitate proteolysis of LpxC by the FtsH protease (40). We previously isolated a spontaneous point mutation in *yciM* that produces a single amino acid substitution in YciM (V43G) that suppresses the mlaA*-dependent hyperproduction of LPS (21). Here we show that this may constitute a gain-of-function mutation that activates the adapter function of YciM to promote the degradation of LpxC (Fig. 5C). Collectively, these data suggest that PldA signaling modulates LPS biosynthesis via modulation of LpxC levels.

### Loss of PldA or FadD does not restore OM defects associated with mlaA* mutant strains.

**FIG 6:**
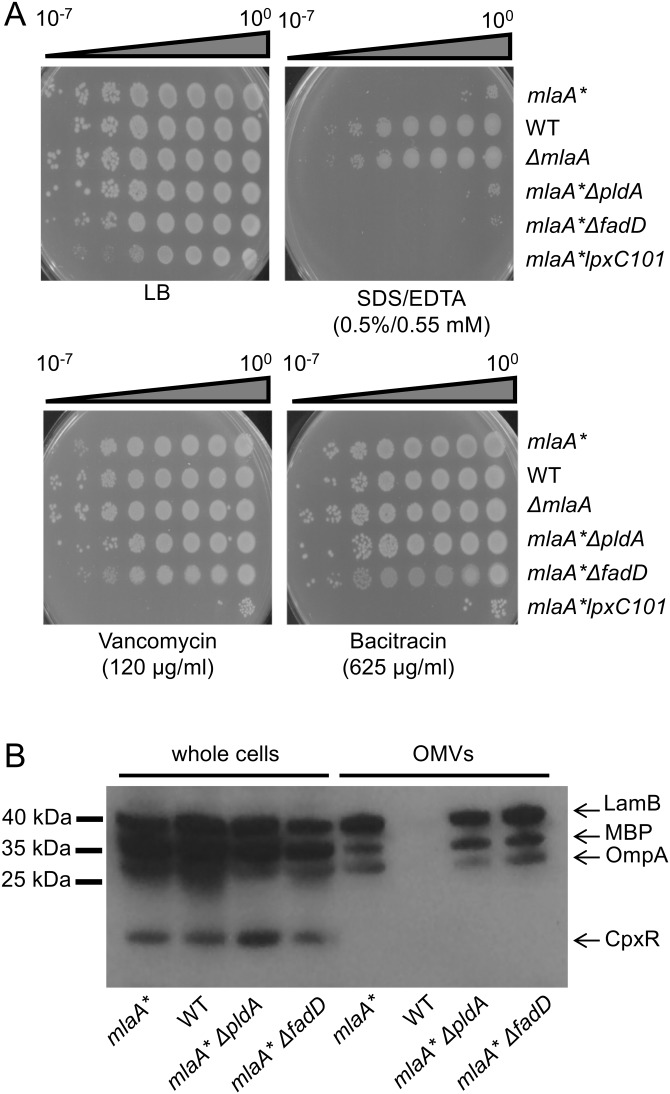
Loss of PldA or FadD does not correct OM permeability or hypervesiculation phenotypes of mlaA* strains. (A) Efficiency-of-plating assays were performed on LB agar plates with the supplements indicated. Ten-fold serial dilutions of cultures were plated as indicated. Loss of *pldA* or *fadD* did not suppress the increased sensitivity of mlaA* cells to a detergent and a chelator (SDS/EDTA). The relative sensitivity of mlaA* cells to large hydrophilic antibiotics was unchanged in the absence of either *pldA* or *fadD*. (B) Lysates from whole cells and OMVs were prepared from equivalent cultures of the strains indicated (normalized by *A*_600_ as described in Materials and Methods) and analyzed by SDS-PAGE and immunoblotting with anti-CpxR, anti-MBP, and anti-LamB/OmpA polyclonal antibodies. Increased amounts of OM and periplasmic material (LamB, OmpA, MBP) in OMV samples indicated that mlaA* cells were hypervesiculated compared to WT cells and that loss of *pldA* or *fadD* did not alter hypervesiculation.

mlaA* mutants exhibit phenotypes consistent with disrupted OM asymmetry, including detergent sensitivity (Fig. 6A) and hypervesiculation (Fig. 6B), but do not exhibit generalized permeability defects that sensitize the cell to large hydrophilic antibiotics, as evident by increased resistance to vancomycin and bacitracin (Fig. 6A). Notably, loss of either PldA or FadD did not restore asymmetry (Fig. 6A) (21). Rather, these mutations simply prevent the increased LPS production induced by PldA activity.

### β-Oxidation (*fadAB*) downstream of FadD was not required for the PldA-dependent increase in LPS production.

**FIG 7:**
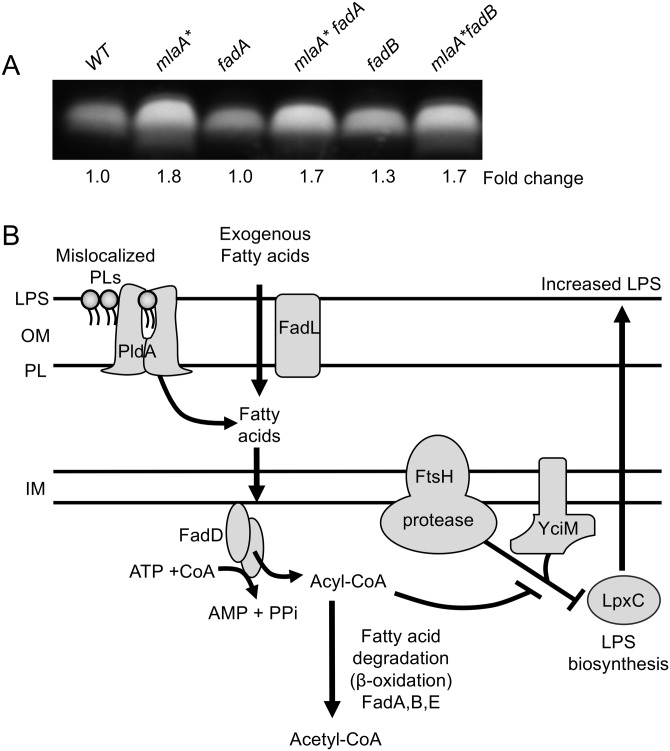
Model of PldA-dependent fatty acid signaling and increased LPS production. (A) Analysis of whole-cell LPS levels indicated that blocking of the β-oxidation pathway via null mutations in either *fadA* or *fadB* did not affect mlaA*-dependent LPS hyperproduction, suggesting that it is acyl-CoA formation that is important for signaling. (B) Model of PldA-dependent fatty acid signaling leading to increased LPS production and maintenance of OM homeostasis. Mislocalized PLs in the outer leaflet of the OM are degraded by the PldA phospholipase, liberating free fatty acids. Exogenous fatty acids can be transported across the OM via FadL. The acyl-CoA synthetase FadD facilitates the uptake and activation of both exogenous and membrane-derived fatty acids across the IM into the cytosol, a process energized by the hydrolysis of ATP. Although the resultant acyl-CoA molecules can be catabolized to acetyl-CoA via a series of reactions (performed by FadA, FadB, and FadE), this β-oxidation pathway is not required for the proposed PldA signaling pathway. Acyl-CoA molecules signal either indirectly or directly to modulate FtsH proteolysis of LpxC, which is facilitated by the adapter protein YciM. This modulatory activity of acyl-CoA leads to decreased proteolysis of LpxC, and the resultant increase in this biosynthetic enzyme acts to increase LPS production.

Upon uptake and activation by FadD in the cytoplasm, fatty acids, can be completely degraded via the β-oxidation cycle, to produce acetyl-CoA that can be used as a carbon and energy source by the cell (30). Notably, disruption of genes that encode key enzymes in this pathway had no effect on mlaA*-dependent LPS hyperproduction (Fig. 7A), suggesting that while the formation of acyl-CoA intermediates is important for PldA signaling, further fatty acid degradation is not required (Fig. 7B).

## DISCUSSION

The gain-of-function mutation mlaA* disrupts OM lipid asymmetry by mislocalizing PLs to the outer leaflet and confers a distinct cell death phenotype due to the stabilization of LpxC and the resulting hyperproduction of LPS (21). We show that loss of either PldA or FadD is sufficient to prevent LPS hyperproduction in response to this disruption of OM asymmetry. While loss of PldA completely suppressed mlaA*-induced cell death, mlaA* fadD double mutants still exhibited some residual cell death. We think it likely that in the absence of FadD, free fatty acids accumulate in mlaA* mutant strains and that this is detrimental to the cell. Indeed, there is evidence to suggest that accumulation of free fatty acids in the cell envelope can disrupt membrane integrity (41). Since LPS levels returned to normal in both the mlaA* fadD and mlaA* pldA mutant strains and *fadD* was epistatic to *pldA*, we conclude that PldA signaling and modulation of LPS production likely occur solely via FadD. Notably, loss of either PldA or FadD does not correct the OM defects in mlaA* mutant cells that can be associated with lipid asymmetry (detergent sensitivity, OM vesiculation). These suppressor mutations do not correct the defect caused by the mlaA* mutation; rather, they prevent the cell’s normal response to it.

Our data suggest that PldA-generated fatty acids in the OM are released into the periplasm and converted to acyl-CoA in the cytoplasm via the acyl-CoA synthetase FadD. Since the subsequent β-oxidation of acyl-CoA is not required for the observed increase in LPS production, acyl-CoA must function in a signaling role to stabilize LpxC (Fig. 7B).

Acyl-CoA already has established roles in the regulation of lipid synthesis through direct modulation of the activities of the transcriptional regulator FadR and the enoyl-acyl carrier protein reductase FabI, which catalyzes the first committed step in the biosynthesis of saturated fatty acids (42, 43). The activity of the FadR transcription regulator is regulated through binding of acyl-CoAs such that it can appropriately coordinate *E. coli* fatty acid metabolism and synthesis in response to fatty acid availability (43, 44). When acyl-CoA levels are high, FadR represses the transcription of genes essential for fatty acid transport, activation, and β-oxidation. Conversely, when acyl-CoA levels are low, FadR can induce the expression of the genes required for the synthesis of unsaturated fatty acids (*fabA* and *fabB*) (43). However, it appears that PldA lipid signaling is unlikely to act solely through FadR since a null mutation in *fadR* (which should mimic a state in which acyl-CoA accumulates) does not phenocopy a mlaA* mutant strain (45–47). Our results suggest an additional signaling role for acyl-CoA in the regulation of LPS levels.

Notably, supplementation of *E. coli* growth medium with exogenous long-chain fatty acids (which are transported into the cell by FadL and converted to acyl-CoA via the fatty acid degradation [FAD] pathway) has been shown to stabilize LpxC levels (23). Furthermore, the activity of FabI, an enoyl-[acyl carrier protein] reductase that catalyzes a committed step in fatty acid biosynthesis, is inhibited by acyl-CoA (42) and that inhibition of FabI also results in stabilization of LpxC levels (23, 37). It has been proposed that in these instances, LpxC stabilization occurs because of decreased flux of substrates into the saturated fatty acid and LPS biosynthetic pathways and that the concentration of the lipid A disaccharide intermediate in LPS biosynthesis acts as a signal to modulate LpxC levels (23). In these instances, the stabilization of LpxC was apparently homeostatic in nature and acted to coordinate PL and LPS biosynthesis. Despite the obvious parallels with our system, where instead of exogenous fatty acids, PldA is generating endogenous fatty acids, it is clear that the constitutive nature and severity of the lipid imbalance in mlaA* strain ultimately lead to a hyperproduction of LPS that disrupts OM homeostasis. However, we propose that in response to transient disturbances of OM asymmetry, e.g., transient exposure to cationic antimicrobial peptides, PldA signaling may indeed play a homeostatic role.

We do not yet know how the production of acyl-CoA in response to PldA activity ultimately leads to the stabilization of LpxC levels. From our present data, it is unclear whether YciM acts downstream of PldA in the signaling pathway or simply that the mutant form of this protein, YciM_V43G_, can act independently to override PldA-dependent LpxC stabilization. The co-occurrence of YciM in members of the family *Enterobacteriaceae*, where regulation of LpxC occurs via FtsH proteolysis, has been previously noted; furthermore, in *Alphaproteobacteria*, where LpxC proteins are degraded by the Lon protease, *yciM* is largely absent (38, 40). Intriguingly, *pldA* is found to co-occur with *yciM* in members of the family *Enterobacteriaceae* and is mostly absent from *Alphaproteobacteria*. It will be of interest to determine whether YciM is integral to our proposed PldA signaling pathway and whether the PldA-dependent upregulation of LPS we observe in *E. coli* plays a role more broadly across the family *Enterobacteriaceae* or even other Gram-negative species.

The OM of Gram-negative bacteria is an organelle at the front line of host-pathogen interactions. In addition to being a permeability barrier that protects against environmental stresses, antimicrobials, and immune system recognition, the OM also functions as a scaffold for a plethora of cell surface virulence factors that determine the outcome of infection. Maintenance of this barrier in the face of various environmental assaults is of the utmost importance. Ultimately, removal of mislocalized PLs from the outer leaflet is just the first step in the maintenance of OM integrity. Ideally, the cell must sense perturbations at this distant organelle and respond accordingly to restore homeostasis. Here we show that PldA phospholipase signaling can fulfill such a role. The utility of membrane-associated phospholipases in relaying messages from the cell surface via lipid second messengers has been well established in eukaryotes (48), and the data presented here suggest that the PldA phospholipase may play an analogous role in bacteria.

## MATERIALS AND METHODS

### Bacterial strains and growth conditions.

All of the strains used in this study (Table S1) were isogenic derivatives of an *araD*^+^ revertant of *E. coli* MC4100 (NR754) (49) and were constructed by generalized P1 transduction or transformation (50). The mlaA* allele was introduced into the NR754 background with a *yfdI*::*kan*-linked marker. Unless otherwise indicated, null alleles were obtained from the Keio collection (51) and Keio alleles were cured of their Kan^r^ cassette with pCP20 as required (52). Strains were grown in Lennox broth (LB) or LB agar at 37°C. LB was supplemented with ampicillin (25 µg/ml), chloramphenicol (20 µg/ml), kanamycin (25 µg/ml), tetracycline (25 µg/ml), vancomycin (120 µg/ml), bacitracin (625 µg/ml), and 0.5% SDS–0.55 mM EDTA as required.

10.1128/mBio.00379-18.1 TABLE S1  Strains and plasmids used in this study. Download TABLE S1, DOCX file, 0.02 MB. Copyright © 2018 May and Silhavy. 2018 May and Silhavy This content is distributed under the terms of the Creative Commons Attribution 4.0 International license.

10.1128/mBio.00379-18.2 TABLE S2  Null mutations that did not suppress mlaA*-dependent cell death. Download TABLE S2, DOCX file, 0.02 MB. Copyright © 2018 May and Silhavy. 2018 May and Silhavy This content is distributed under the terms of the Creative Commons Attribution 4.0 International license.

### Plasmid construction.

The plasmids used in this study are listed in Table S1. To construct pBBR1-*fadD*, the *fadD* open reading frame and a 220-bp region directly upstream of the *fadD* gene were PCR amplified with oligonucleotides KM107_fadD_SacI_F (AATTGAGCTCAGTTGTAACTGAATAATTGC) and KM108_fadD_KpnI_R (AATTGGTACCTCATCAGGCTTTATTGTCCAC). The amplicon was then SacI-KpnI digested and cloned into similarly digested pBBR1MCS (53).

### Spent-medium transition assay.

Spent medium was prepared by culturing WT bacterial cells for 24 h in LB at 37°C with aeration. Cells were pelleted by centrifugation, and the supernatant was collected and filtered through a 0.2-μm filter (Millipore) to eliminate cells and large cellular debris. This filtered supernatant was used as spent medium. For cell death assays, overnight cultures grown at 37°C were diluted in fresh LB and grown at 37°C with shaking until late exponential phase (optical density at 600 nm [OD_600_ ] of ~0.8). Cultures were pelleted by centrifugation and resuspended in spent medium to induce the transition into stationary phase. Cultures were transferred to 24-well plates, sealed with breathable film, and incubated with aeration at 37°C. Cell density (*A*_600_) was monitored for up to 16 h.

### LPS analyses.

LPS levels were analyzed as previously described (21). Briefly, 5 × 10^8^ cells from an overnight culture (16 h) were collected by centrifugation and resuspended in 100 μl of 1× LDS sample buffer (Life Sciences) plus 4% β-mercaptoethanol. Samples were denatured at 100°C for 10 min, allowed to cool, and then treated with 125 ng/μl proteinase K (New England Biolabs) at 55°C for 16 h. Proteinase K was heat inactivated, and the lysates were resolved by SDS-PAGE (NuPAGE 4 to 12% Bis-Tris Gradient Gels; Life Sciences). Gels were stained with the Pro-Q Emerald 300 LPS Gel Stain kit (Molecular Probes) in accordance with the manufacturer’s instructions. LPS bands were visualized by UV transillumination, and relative band intensities were determined with the Quantity One imaging software (Bio-Rad).

### Sensitivity assays.

Efficiency-of-plating assays were used to determine the relative sensitivities of strains to antibiotics (vancomycin and bacitracin), a detergent (SDS), and a chelator (EDTA). Assays were performed by preparing serial dilutions (10-fold) of overnight cultures (standardized by OD_600_), replica plating them onto LB agar and selective medium, and then incubating plates overnight at 37°C.

### OMV preparation.

To isolate OM vesicles (OMVs), cells were grown in LB to mid-exponential phase (OD_600_ of ~0.6 to 0.8). Samples of 5 × 10^8^ cells were collected for whole-cell lysate controls. For OMVs, cultures equivalent to 1.5 × 10^9^ cells were centrifuged (5,000 × *g* for 5 min at room temperature) and the supernatants were collected and adjusted to equal volumes with fresh LB. Supernatants were filtered through 0.2-μm filters to eliminate whole cells and large cellular debris. This filtered supernatant was then subjected to a second round of filtration through an Amicon Ultra-15 centrifugal filter (Millipore) with a 100,000 molecular weight cutoff to isolate and concentrate OMVs. Samples obtained with this filter were resuspended in 50 μl of SDS-PAGE buffer and boiled for 10 min, and 10 μl of each sample (equivalent to supernatant from 3 × 10^8^ cells) was resolved by SDS-PAGE and analyzed by Western immunoblotting.

### Whole-cell lysate preparation.

Cultures were grown overnight and then subcultured 1:100 in fresh LB. Subcultures were grown at 37°C for 2 h (*A*_600_ of ~0.5 to 0.6), and the equivalent of 5 × 10^8^ cells (1 ml of cells at an *A*_600_ of 1.0) was pelleted by centrifugation (10,000 × *g* for 5 min) and solubilized in 100 μl of LDS buffer (Life Sciences) at 100°C for 5 to 10 min. A 10-μl volume of each sample (equivalent to 5 × 10^7^ cells) was analyzed by SDS-PAGE and immunoblotting.

### Immunoblot analyses.

OMV or whole-cell lysate samples were resolved by 12% SDS-PAGE (Bis-NuPAGE MES [morpholinepropanesulfonic acid] buffer), transferred to a 0.2-µm-pore-size nitrocellulose membrane, and probed as indicated with the following dilutions of rabbit polyclonal antibodies (from our laboratory collection of antisera raised against denatured proteins): anti-CpxR antibody, 1:30,000; anti-MBP antibody, 1:30,000; anti-LamB antibody (with cross-reactivity to OmpA), 1:30,000. Anti-LpxC antibody (1:5,000) (36) was provided by Katherine Young (Merck Research Laboratories). Membranes were subsequently probed with a donkey anti-rabbit secondary antibody conjugated to horseradish peroxidase and incubated with enhanced chemiluminescence substrate (Millipore Classico). The ECL signal was captured by X-ray film.

## Note Added after Publication

In the originally published version, an incorrect definition was introduced for the FAD pathway. The article was corrected online on 25 April 2018.
