# Erratum for May and Silhavy, “The Escherichia coli Phospholipase PldA Regulates Outer Membrane Homeostasis via Lipid Signaling”

**DOI:** 10.1128/mBio.00718-18

**Published:** 2018-05-01

**Authors:** Kerrie L. May, Thomas J. Silhavy

**Affiliations:** a Department of Molecular Biology, Princeton University, Lewis Thomas Laboratory, Princeton, New Jersey, USA

## ERRATUM

Volume 9, issue 2, e00379-18, 2018, https://doi.org/10.1128/mBio.00379-18. On PDF page 10, an incorrect definition was introduced for the FAD pathway. The sentence should read as shown below. The original article has been corrected online.

Notably, supplementation of E. coli growth medium with exogenous long-chain fatty acids (which are transported into the cell by FadL and converted to acyl-CoA via the fatty acid degradation [FAD] pathway) has been shown to stabilize LpxC levels (23).

