# Supplementary material for: The Escherichia coli Phospholipase PldA Regulates Outer Membrane Homeostasis via Lipid Signaling
Source: mBio. 2018 Mar 20;9(2):e00379-18. doi: 10.1128/mBio.00379-18 (PMC5874903; doi:10.1128/mBio.00379-18)
Supplement: TABLE S1 [file mbo002183797st1.docx]

**Table S1. Strains and plasmids used in this study.**

| **Strain** | **Genotype** | **Reference** |
| --- | --- | --- |
| MC4100 | F-  *[araD139]_B/r_*, *Δ(argF-lac)169*, *λ^-^*, *e14*, *flhD5301*,  *Δ(fruK-yeiR)725(fruA25)*, *relA1*, *rpsL150*(strR),  *rbsR22*, *Δ(fimB-fimE)632(::IS1)*, *deoC1* | (1) |
| NR754 | MC4100 ara^+^ | (2) |
| HC735 | MC4100 ara^+^ Δ*yfdI* | (3) |
| HC687 | MC4100 ara^+^ Δ*yfdI mlaA** | (3) |
| HC726 | MC4100 ara^+^ Δ*yfdI* *mlaA** *pldA*::*kan* | (3) |
| HC736 | MC4100 ara^+^ Δ*yfdI* Δ*mlaA* | (3) |
| HC1247 | MC4100 ara^+^ Δ*yfdI mlaA** *yciM*^V43G^ *ycjM*::Tn*10* | (3) |
| HC1390 | MC4100 ara^+^ Δ*yfdI mlaA** *lpxC101* *leuB*::Tn*10* | (3) |
| KM33 | MC4100 ara^+^ Δ*yfdI* *mlaA** *bla P*_ara_::*yjgPQ* | (4) & this study |
| KM34 | MC4100 ara^+^ Δ*yfdI* *bla P*_ara_::*yjgPQ* | (4) & this study |
| KM195 | MC4100 ara^+^ Δ*yfdI mlaA* fadD::kan* | This study |
| KM196 | MC4100 ara^+^ Δ*yfdI fadD::kan* | This study |
| KM238 | MC4100 ara+ *ΔyfdI* mlaA* *ΔpldA* *fadD::kan* | This study |
| KM131 | MC4100 ara^+^ Δ*yfdI mlaA*pldB::kan* | This study |
| KM132 | MC4100 ara^+^ Δ*yfdI mlaA*tesA::kan* | This study |
| KM136 | MC4100 ara^+^ Δ*yfdI mlaA*glpT::kan* | This study |
| KM137 | MC4100 ara^+^ Δ*yfdI mlaA*glpQ::kan* | This study |
| KM138 | MC4100 ara^+^ Δ*yfdI mlaA*lplT::kan* | This study |
| KM173 | MC4100 ara^+^ Δ*yfdI* Δ*fadL* *p*ZS21*-mlaA** | This study |
| KM174 | MC4100 ara^+^ Δ*yfdI* Δ*fadL p*ZS21*-mlaA* | This study |
| KM232 | MC4100 ara^+^ Δ*yfdI mlaA* tsp::kan* | This study |
| KM327 | MC4100 ara^+^ Δ*yfdI mlaA* aas::kan* | This study |
| KM439 | MC4100 ara^+^ Δ*yfdI fadA::kan* | This study |
| KM440 | MC4100 ara^+^ Δ*yfdI mlaA*fadA::kan* | This study |
| KM441 | MC4100 ara^+^ Δ*yfdI fadB::kan* | This study |
| KM442 | MC4100 ara^+^ Δ*yfdI mlaA*fadB::kan* | This study |
| KM495 | MC4100 ara^+^ Δ*yfdI mlaA* fadD::kan*  pBBR1-*fadD* | This study |
| **Plasmid** | **Relevant features** | **Reference** |
| pBBR1MCS | Broad-host range vector; *Cm^R^* | (5) |
| pBBR1-*fadD* | *fadD* ORF & 220 bp upstream cloned into pBBR1MCS | This study |
| *p*ZS21*-mlaA** | *mlaA** cloned into pZS21 low-copy vector (Km^R^) | (3) |
| *p*ZS21*-mlaA* | *mlaA* cloned into pZS21 low-copy vector (Km^R^) | (3) |

**Supplemental References**

1. **Casadaban MJ**. 1976. Transposition and fusion of the lac genes to selected promoters in Escherichia coli using bacteriophage lambda and Mu. J Mol Biol **104**:541–555.

2. **Button JE**, **Silhavy TJ**, **Ruiz N**. 2007. A suppressor of cell death caused by the loss of sigmaE downregulates extracytoplasmic stress responses and outer membrane vesicle production in Escherichia coli. J Bacteriol **189**:1523–1530.

3. **Sutterlin HA**, **Shi H**, **May KL**, **Miguel A**, **Khare S**, **Huang KC**, **Silhavy TJ**. 2016. Disruption of lipid homeostasis in the Gram-negative cell envelope activates a novel cell death pathway. Proc Natl Acad Sci USA **113**:E1565–74.

4. **Yao Z**, **Davis RM**, **Kishony R**, **Kahne D**, **Ruiz N**. 2012. Regulation of cell size in response to nutrient availability by fatty acid biosynthesis in Escherichia coli. Proc Natl Acad Sci USA **109**:E2561–8.

5. **Kovach ME**, **Phillips RW**, **Elzer PH**, **Roop RM**, **Peterson KM**. 1994. pBBR1MCS: a broad-host-range cloning vector. BioTechniques **16**:800–802.

6. **Baba T**, **Ara T**, **Hasegawa M**, **Takai Y**, **Okumura Y**, **Baba M**, **Datsenko KA**, **Tomita M**, **Wanner BL**, **Mori H**. 2006. Construction of Escherichia coli K-12 in-frame, single-gene knockout mutants: the Keio collection. Molecular Systems Biology **2**:–.

7. **Nunn WD**, **Simons RW**. 1978. Transport of long-chain fatty acids by Escherichia coli: mapping and characterization of mutants in the fadL gene. PNAS **75**:3377–3381.

8. **Hearn EM**, **Patel DR**, **Lepore BW**, **Indic M**, **van den Berg B**. 2009. Transmembrane passage of hydrophobic compounds through a protein channel wall. Nature **458**:367–370.

9. **Soltes GR**, **Martin NR**, **Park E**, **Sutterlin HA**, **Silhavy TJ**. 2017. Distinctive Roles for Periplasmic Proteases in the Maintenance of Essential Outer Membrane Protein Assembly. J Bacteriol **199**:e00418–17.

10. **Azizan A**, **Black PN**. 1994. Use of transposon TnphoA to identify genes for cell envelope proteins of Escherichia coli required for long-chain fatty acid transport: the periplasmic protein Tsp potentiates long-chain fatty acid transport. J Bacteriol **176**:6653–6662.

11. **Larson TJ**, **Ehrmann M**, **Boos W**. 1983. Periplasmic glycerophosphodiester phosphodiesterase of Escherichia coli, a new enzyme of the glp regulon. J Biol Chem **258**:5428–5432.

12. **Larson TJ**, **Schumacher G**, **Boos W**. 1982. Identification of the glpT-encoded sn-glycerol-3-phosphate permease of Escherichia coli, an oligomeric integral membrane protein. J Bacteriol **152**:1008–1021.

13. **Harvat EM**, **Zhang Y-M**, **Tran CV**, **Zhang Z**, **Frank MW**, **Rock CO**, **Saier MH**. 2005. Lysophospholipid flipping across the Escherichia coli inner membrane catalyzed by a transporter (LplT) belonging to the major facilitator superfamily. J Biol Chem **280**:12028–12034.

14. **Jackowski S**, **Jackson PD**, **Rock CO**. 1994. Sequence and function of the aas gene in Escherichia coli. J Biol Chem **269**:2921–2928.

15. **Karasawa K**, **Kudo I**, **Kobayashi T**, **Sa-Eki T**, **Inoue K**, **Nojima S**. 1985. Purification and characterization of lysophospholipase L2 of Escherichia coli K-12. J Biochem **98**:1117–1125.

16. **Hsu L**, **Jackowski S**, **Rock CO**. 1991. Isolation and characterization of Escherichia coli K-12 mutants lacking both 2-acyl-glycerophosphoethanolamine acyltransferase and acyl-acyl carrier protein synthetase activity. J Biol Chem **266**:13783–13788.

17. **Karasawa K**, **Yokoyama K**, **Setaka M**, **Nojima S**. 1999. The Escherichia coli pldC gene encoding lysophospholipase L(1) is identical to the apeA and tesA genes encoding protease I and thioesterase I, respectively. J Biochem **126**:445–448.

18. **Yao J**, **Rock CO**. 2017. Exogenous fatty acid metabolism in bacteria. Biochimie **141**:30–39.
