# Supplementary material for: The Escherichia coli Phospholipase PldA Regulates Outer Membrane Homeostasis via Lipid Signaling
Source: mBio. 2018 Mar 20;9(2):e00379-18. doi: 10.1128/mBio.00379-18 (PMC5874903; doi:10.1128/mBio.00379-18)
Supplement: TABLE S2 [file mbo002183797st2.docx]

**Table S2. Null-mutations that did not suppress *mlaA**-dependent cell death^I^**

| **Target gene** | **Function of gene product** | | **Reference** |
| --- | --- | --- | --- |
| *fadL^II^* | transport of exogenous fatty acids across the OM | (6-8) | |
| *tsp* | potentiates transport of long chain fatty acids | (9, 10) | |
| *glpQ* | glycerophosphodiester phosphodiesterase | (6, 11) | |
| *glpT* | sn-glycerol 3-phosphate:phosphate antiporter | (6, 12) | |
| *lplT* | lysophospholipid transporter | (6, 13) | |
| *aas* | Uptake of exogenous 2-acyllysophospholipids | (6, 14) | |
| *pldB* | lysophospholipase | (6, 15, 16) | |
| *tesA* | multifunctional esterase | (6, 17) | |
| *fadA* | degradation of fatty acids via the β-oxidation cycle | (18) | |
| *fadB* | degradation of fatty acids via the β-oxidation cycle | (18) | |

^I^ stationary-phase cell death as determined by A_600_ of overnight culture

^II^ *fadL* tested in *mlaA*^+^/*mlaA** diploid

**Supplemental References**

1. **Casadaban MJ**. 1976. Transposition and fusion of the lac genes to selected promoters in Escherichia coli using bacteriophage lambda and Mu. J Mol Biol **104**:541–555.

2. **Button JE**, **Silhavy TJ**, **Ruiz N**. 2007. A suppressor of cell death caused by the loss of sigmaE downregulates extracytoplasmic stress responses and outer membrane vesicle production in Escherichia coli. J Bacteriol **189**:1523–1530.

3. **Sutterlin HA**, **Shi H**, **May KL**, **Miguel A**, **Khare S**, **Huang KC**, **Silhavy TJ**. 2016. Disruption of lipid homeostasis in the Gram-negative cell envelope activates a novel cell death pathway. Proc Natl Acad Sci USA **113**:E1565–74.

4. **Yao Z**, **Davis RM**, **Kishony R**, **Kahne D**, **Ruiz N**. 2012. Regulation of cell size in response to nutrient availability by fatty acid biosynthesis in Escherichia coli. Proc Natl Acad Sci USA **109**:E2561–8.

5. **Kovach ME**, **Phillips RW**, **Elzer PH**, **Roop RM**, **Peterson KM**. 1994. pBBR1MCS: a broad-host-range cloning vector. BioTechniques **16**:800–802.

6. **Baba T**, **Ara T**, **Hasegawa M**, **Takai Y**, **Okumura Y**, **Baba M**, **Datsenko KA**, **Tomita M**, **Wanner BL**, **Mori H**. 2006. Construction of Escherichia coli K-12 in-frame, single-gene knockout mutants: the Keio collection. Molecular Systems Biology **2**:–.

7. **Nunn WD**, **Simons RW**. 1978. Transport of long-chain fatty acids by Escherichia coli: mapping and characterization of mutants in the fadL gene. PNAS **75**:3377–3381.

8. **Hearn EM**, **Patel DR**, **Lepore BW**, **Indic M**, **van den Berg B**. 2009. Transmembrane passage of hydrophobic compounds through a protein channel wall. Nature **458**:367–370.

9. **Soltes GR**, **Martin NR**, **Park E**, **Sutterlin HA**, **Silhavy TJ**. 2017. Distinctive Roles for Periplasmic Proteases in the Maintenance of Essential Outer Membrane Protein Assembly. J Bacteriol **199**:e00418–17.

10. **Azizan A**, **Black PN**. 1994. Use of transposon TnphoA to identify genes for cell envelope proteins of Escherichia coli required for long-chain fatty acid transport: the periplasmic protein Tsp potentiates long-chain fatty acid transport. J Bacteriol **176**:6653–6662.

11. **Larson TJ**, **Ehrmann M**, **Boos W**. 1983. Periplasmic glycerophosphodiester phosphodiesterase of Escherichia coli, a new enzyme of the glp regulon. J Biol Chem **258**:5428–5432.

12. **Larson TJ**, **Schumacher G**, **Boos W**. 1982. Identification of the glpT-encoded sn-glycerol-3-phosphate permease of Escherichia coli, an oligomeric integral membrane protein. J Bacteriol **152**:1008–1021.

13. **Harvat EM**, **Zhang Y-M**, **Tran CV**, **Zhang Z**, **Frank MW**, **Rock CO**, **Saier MH**. 2005. Lysophospholipid flipping across the Escherichia coli inner membrane catalyzed by a transporter (LplT) belonging to the major facilitator superfamily. J Biol Chem **280**:12028–12034.

14. **Jackowski S**, **Jackson PD**, **Rock CO**. 1994. Sequence and function of the aas gene in Escherichia coli. J Biol Chem **269**:2921–2928.

15. **Karasawa K**, **Kudo I**, **Kobayashi T**, **Sa-Eki T**, **Inoue K**, **Nojima S**. 1985. Purification and characterization of lysophospholipase L2 of Escherichia coli K-12. J Biochem **98**:1117–1125.

16. **Hsu L**, **Jackowski S**, **Rock CO**. 1991. Isolation and characterization of Escherichia coli K-12 mutants lacking both 2-acyl-glycerophosphoethanolamine acyltransferase and acyl-acyl carrier protein synthetase activity. J Biol Chem **266**:13783–13788.

17. **Karasawa K**, **Yokoyama K**, **Setaka M**, **Nojima S**. 1999. The Escherichia coli pldC gene encoding lysophospholipase L(1) is identical to the apeA and tesA genes encoding protease I and thioesterase I, respectively. J Biochem **126**:445–448.

18. **Yao J**, **Rock CO**. 2017. Exogenous fatty acid metabolism in bacteria. Biochimie **141**:30–39.
